# Evaluation of the effects of ascorbic acid on metabolism of human mesenchymal stem cells

**DOI:** 10.1186/s13287-018-0825-1

**Published:** 2018-04-06

**Authors:** Koichi Fujisawa, Kazusa Hara, Taro Takami, Sae Okada, Toshihiko Matsumoto, Naoki Yamamoto, Isao Sakaida

**Affiliations:** 1 Center for Regenerative Medicine, Yamaguchi University School of Medicine, Minami Kogushi 1-1-1, Ube, Yamaguchi, 755-8505 Japan; 2 Department of Gastroenterology and Hepatology, Yamaguchi University Graduate School of Medicine, Minami Kogushi 1-1-1, Ube, Yamaguchi, 755-8505 Japan

**Keywords:** Ascorbic acid, Mesenchymal stem cell, Meztabolome, Transcriptome, Extracellular matrix, Hypoxia

## Abstract

**Background:**

Mesenchymal stem cells (MSCs) are multipotent cells holding much promise for applications in regenerative medicine. However, with problems such as aging, increases in heteroploid cells, genomic instability, and reduced maintenance of stemness, more stable culturing methods and the production of MSCs with an improved therapeutic effect are desired. Ascorbic acid (AsA), which is a cofactor for a variety of enzymes and has an antioxidant effect, cannot be synthesized by certain animals, including humans. Nevertheless, little attention has been paid to AsA when culturing MSCs.

**Methods:**

We analyzed the effect of adding AsA to the culture medium on the proliferation and metabolism of human MSCs by serial analysis of gene expression and metabolome analysis.

**Results:**

We found that AsA promotes MSC proliferation, and is particularly useful when expanding MSCs isolated from bone marrow. Serial analysis of gene expression and metabolome analysis suggested that, due to HIF1α accumulation caused by decreased activity of the enzymes that use AsA as a coenzyme in cultures without AsA, genes downstream of HIF1α are expressed and there is a conversion to a hypoxia-mimetic metabolism. AsA promotes HIF1α breakdown and activates mitochondria, affecting cell proliferation and metabolism. Comprehensive evaluation of the effects of AsA on various metabolic products in MSCs revealed that AsA increases HIF1α hydroxylase activity, suppressing HIF1a transcription and leading to mitochondrial activation.

**Conclusions:**

Adding AsA during MSC expansion leads to more efficient preparation of cells. These are expected to be important findings for the future application of MSCs in regenerative medicine.

**Electronic supplementary material:**

The online version of this article (10.1186/s13287-018-0825-1) contains supplementary material, which is available to authorized users.

## Background

Mesenchymal stem cells (MSCs) have the ability to differentiate into mesenchymal cells such as fat cells, bone cells, and cartilage cells, and they hold promise for applications in regenerative medicine. Bone marrow-derived MSCs in particular are used in a variety of treatments since it is known that they exhibit multipotency, suppress inflammation, etc. To date, our research focused on the applicability of MSCs for the treatment of liver cirrhosis, and we previously reported that MSCs ameliorate thioacetamide-induced oxidative stress in hepatocytes both in vivo and in vitro [1]. However, it is conceivable that the therapeutic quality of the MSCs used differs depending on the culture conditions. The oxygen concentration in the bone marrow is considered to be in the order of 1–5% [2], and it has been reported that culturing in a 20% oxygen environment could cause stress and impair the maintenance of genetic stability. In addition, repeated culturing causes aging and problems such as an increase in heteroploid cells, reduced maintenance of stemness, and genomic instability. However, the mechanisms underlying these problems remain largely unclear; thus, there is a need to obtain basic knowledge regarding culturing MSCs with an improved therapeutic effect by investigating culturing conditions and chemical treatments.

Ascorbic acid (AsA), an organic compound with a lactone structure, has long been widely studied and is known to have multiple physiological functions: it eliminates reactive oxygen species caused by oxidative stress, is involved in cell division, and suppresses aging. We have previously reported on the relationship between AsA oxidation and cell proliferation [3], as well as on the important effect that the protein regucalcin, which is involved in AsA metabolism, has on aging and hepatic carcinoma [4]. Studies on cell proliferation have reported that AsA has an effect on the ability of cells to proliferate and differentiate [5, 6]. The mechanisms by which AsA is involved in proliferation and differentiation involve a number of factors; apart from its antioxidative effects, AsA participates as a cofactor in a variety of enzymatic reactions. Nevertheless, much remains unknown about the detailed mechanisms of action of this molecule. In previous stem cell studies, AsA has been reported to induce iPS cell differentiation [7] and to promote differentiation from ES and iPS cells into cardiomyocytes [8, 9]. Furthermore, AsA is considered to be involved in collagen synthesis promotion, the MEK-ERK1/2 pathway, NADPH oxidase (NOX), and endothelial NO synthase (eNOS) [10].

Some animals, such as humans and primates, are unable to synthesize AsA because of loss of activity of the enzyme l-gulono-gamma-lactone-oxidase, which catalyzes the final step in the AsA synthesis pathway. AsA is added to some specialized culture media and has been reported to be effective for differentiating MSCs into bone tissue when added to the culture medium. However, in general, when culturing human MSCs (hMSCs), Dulbecco’s modified Eagle’s medium (DMEM), a culture medium that does not contain AsA, supplemented with 10% fetal bovine serum (FBS) and antibiotics, is commonly used. DMEM does not contain AsA because it was originally developed for culturing cells that can synthesize AsA, such as mouse cells. In addition, AsA is unstable in aqueous solutions; thus, FBS-derived AsA potentially is deactivated to a significant degree, and these culturing methods are likely AsA-deficient. In this study, we added l-ascorbic acid 2-phosphate (AAP), which is an AsA derivative that is stable in culture, to DMEM with 10% FBS, and we analyzed the proliferative capacity, extracellular matrix production, gene expression using serial analysis of gene expression (SAGE), and metabolic products using metabolome analysis. Currently, no reports on the effects of AsA addition on MSCs using comprehensive analysis of metabolic products are available. Thus, we expected the findings from this study to benefit the field of regenerative medicine.

## Methods

### Cells and cell culture

MSCs were derived from bone marrow cells (AllCells, Alameda, CA、USA) and were used at passage (P)4. Mononuclear cells (MNCs) were purchased from Lonza, Tokyo, Japan. Flow cytometry (Gallios™, Beckman Coulter, Tokyo, Japan) was used to confirm that the MSCs were CD45^−^CD11b^−^ CD90^+^CD73^+^CD105^+^.

### Proliferation assay

MSCs were seeded into each well of a 96-well plate (3000 cells/well), and AAP (Tokyo Chemical Industries, Ltd. Tokyo, Japan) was added at indicated concentrations. Proliferation was measured using the IncuCyte HD Imaging System (Essen BioScience, Tokyo, Japan).

### Apoptosis assay

Aliquots of 3 × 10^3^ cells were seeded in 96-well plates, AAP was added, and the cells were cultured for 1 day. Doxorubicin was then added, and a CyQuant assay was performed to measure the number of cells 24 h later. A Caspase 3 Assay Kit (Promega, Madison, WI, USA) was then used to measure caspase 3 activity, and the activity per cell was calculated. The experiments were performed in triplicate with n = 6.

### Differentiation ability assay

A Human Mesenchymal Stem Cell Functional Identification Kit was purchased from R&D Systems (Minneapolis, MN, USA). Cell differentiation was performed following the manufacturer’s instructions. Briefly, cells were seeded at a density of 2 × 10^4^ cells/cm2. When the cells were 100% confluent, the media in each well were replaced with 0.5 mL of Adipogenic Differentiation Media to induce adipogenesis. The media were replaced with fresh Adipogenic Differentiation Media every 3–4 days. After 5–7 days, we confirmed that lipid vacuoles started to appear in the induced cells. After 14 days, the cells were stained with Oil Red O. The total cell number and adipogenic cell number were counted in the ×20 field for each AAP concentration (*n* = 6) (Additional file 1).

### Collagen quantification

Collagen produced by cultured cells was stained using the Sirius Red/Fast Green Collagen Staining Kit. After washing, the cells were photographed under a microscope, and the light absorption at 540 nm (Sirius Red) and 605 nm (Fast Green) were measured to infer the amount of collagen and the amount of non-collagen proteins, respectively.

### Western blot analysis

Protein lysates were obtained by homogenizing tissues or cell pellets in sample buffer containing 62.5 mM Tris-HCl (pH 6.8), 4% SDS, 200 mM dithiothreitol, 10% glycerol, and 0.001% bromophenol blue at a ratio of 1:10 (w*/*v), followed by boiling. Western blot analysis was performed with purified polyclonal anti-human rabbit IgG. Antibodies against beta-actin (Sigma-Aldrich, St. Louis, MO, USA), were purchased from the indicated suppliers.

### SAGE analysis

The total RNA was isolated from cells using TRIzol Reagent (Life Technologies, Carlsbad, CA, USA). Ion Ampliseq Transcriptome Human Gene Expression Kit (Life Technologies) was used for library creation. An Ion Proton next-generation sequencer library of analysis beads was created, and an Ion PI IC 200 Kit (Life Technologies) and an Ion PI Chip Kit v2 BC were used for sequencing, using an Ion Proton next-generation sequencer. The results of metabolomic analysis and SAGE were integrated by Ingenuity Pathways Analysis (IPA).

### Metabolome analysis

The culture medium was aspirated from a 10-cm cell culture dish and the cells were washed twice with 5% mannitol solution (10 mL, followed by 2 mL). The cells were then treated with 800 μL methanol and left at rest for 30 s in order to inactivate enzymes. Next, the cell extract was treated with 550 μL Milli-Q water containing internal standards (H3304–1002, Human Metabolome Technologies, Inc., Tsuruoka, Japan) and left at rest for another 30 s. The extract was obtained and centrifuged at 2300 × g and 4 °C for 5 min; 800 μL of the upper aqueous layer was then centrifugally filtered through a Millipore 5-kDa cutoff filter at 9100 × g and 4 °C for 120 min to remove proteins. The filtrate was centrifugally concentrated and resuspended in 50 μL of Milli-Q water for capillary electrophoresis time-of-flight mass spectrometry (CE-TOFMS) analysis.

### Measurement of oxygen consumption rate (OCR)

OCR measurements were performed using a Seahorse Biosciences XF96 Extracellular Flux Analyzer. Cells were seeded at 6000 cells/well in XF96 microplates (Seahorse Biosciences North Billerica, MA, USA). After a 24-h incubation, the growth media were exchanged for XF Assay Medium (Seahorse Biosciences) supplemented with 25 mM glucose. OCR measurements were made over 5-min periods following a 3-min mix period. Cells were treated by sequential addition of 1 μg/mL oligomycin, 300 nM xcarbonylcyanide-p-trifluoromethoxyphenylhydrazone (FCCP), and 2 μM rotenone. The spare respiratory capacity and coupling efficiency were calculated according to the Seahorse Bioscience instructions and the basal OCR was normalized to the cell number.

### Statistical analysis

The results were analyzed by either the Student’s *t* test or Welsh’s two-factor *t* tests. Analysis of variance (ANOVA) with post hoc analysis using Turkey’s multiple comparison test was used for comparisons between multiple groups. The data are presented as the mean ± standard deviation, with significance level established at *p* < 0.05.

## Results

### AsA promotes MSC proliferation and promotes MSC expansion from MNCs

**Fig. 1:**
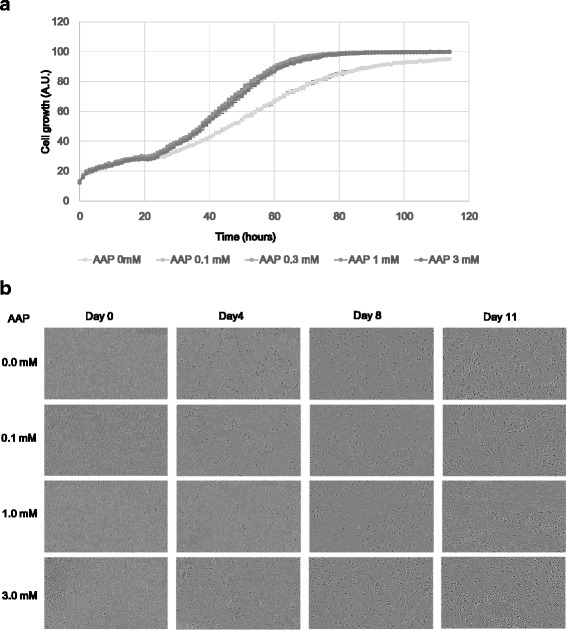
Effect of AsA on MSC proliferation. **a** Time-course analysis of the effect of AsA on proliferation. AAP was added at P4 MSC seeding, and IncuCyte was used to evaluate proliferation in terms of cell area. **b** Promotive effect of AsA on proliferation during expansion (P0) of MNCs obtained from bone marrow. MNCs from which erythrocytes were eliminated after collection from the bone marrow were purchased, and AAP was added at the time of seeding. MSCs with a spindle apparatus are indicated with *arrows*

To investigate the effect of AsA on MSC proliferation, we evaluated proliferation after adding 0, 0.1, 0.3, 1, or 3 mM of AAP to the culture medium (the serum concentration is 0.03–0.1 mM, approximately), using MSCs at P4. The addition of AAP clearly dose-dependently promoted cell proliferation at 24 h post seeding, without a significant difference between the concentrations tested (Fig. 1a). In addition, when cultured past 100% confluency, the cells cultured with AAP tended to form multiple layers, culminating in the cells detaching from the dish and forming a close sheet (data not shown). When MSCs are expanded from bone marrow-derived MNCs (BMMNCs) from which hematocytes have been eliminated, proliferation takes a long time because of the very low percentage of MSCs. Thus, we evaluated the effect of adding AAP to the medium on proliferation when culturing MSCs from BMMNCs. On day 0, only MNCs were observed. However, by day 8, marked proliferation of MSCs was observed in the AAP-supplemented groups, and on day 11, MSC proliferation was clearly promoted in the AAP-supplemented groups (Fig. 1b).

### AsA promotes extracellular matrix synthesis by MSCs

**Fig. 2:**
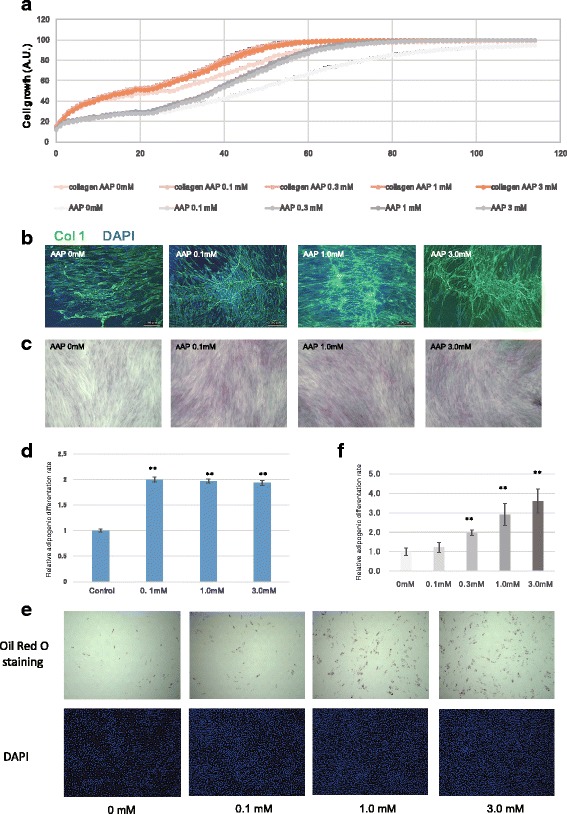
Effects of AsA on collagen maturation and differentiation in MSCs. **a** Proliferation of cells in collagen-coated dishes (*orange line*) and non-coated polystyrene dish (*gray line*). Data for the latter are the same data as those in Fig. 1a. **b** IHC of Col1. Col1 antibody: *green*, DAPI: *blue*. **c** Sirius Red/Fast Green staining for collagen. 100% confluent cells were used for staining. **d** Quantification of total collagen. After staining, the amounts of collagen and non-collagen proteins were inferred by measuring the absorption at 540 nm (Sirius Red) and 605 nm (Fast Green). **e** Adipogenic differentiation staining. After culturing the cells to 100% confluence, the culture medium was replaced with adipogenic differentiation medium. After 7 days of culturing, lipids were stained with Oil Red O. In the *bottom row*, nuclei are counterstained with DAPI. **f** Quantitative evaluation of adipogenic differentiation. The relative adipogenic differentiation rate was expressed as the number of cells that underwent adipogenic differentiation divided by the total number of cells at an AAP of 0 mM

AsA is regarded to be involved in increased higher-order structure and to enhance the extracellular matrix. In the absence of AAP, proliferation was higher in collagen-coated plates than in non-coated plates at 24 h. Addition of AAP further enhanced proliferation at 24 h (Fig. 2a). Addition of AAP in a polystyrene culture plate resulted in nearly the same cell count at 60 h after seeding as that observed in the collagen-coated dish without AAP addition. Immunohistostaining revealed a marked increase in extracellular collagen 1 fibers in cells cultured with AAP (Fig. 2b), which was confirmed by quantitative analysis using Sirius Red staining (Fig. 2c). No significant difference in the amount of collagen was seen for the different AAP concentrations (Fig. 2d).

### AsA promotes adipogenic differentiation of MSCs

As it has been reported that AsA can promote cell differentiation, we evaluated its effect on MSCs. Adipogenic differentiation was promoted in a concentration-dependent manner (Fig. 2e, f). Differentiation potential of BMSCs for osteogenic, cholangiogenic, and myogenic with and without addition of AAP (1 mM) by real-time PCR was also evaluated (Additional file 2: Figure S2).

### AsA upregulates the tricarboxylic acid (TCA) cycle

**Fig. 3:**
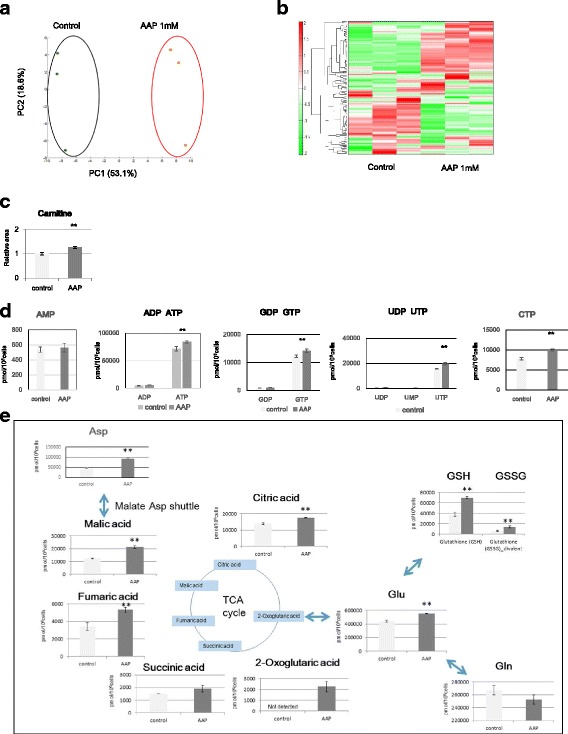
Metabolome analysis and SAGE. **a** PCA of normalized metabolic data obtained from MSCs 48 h after AAP addition and controls (0 mM). Percentage values indicated on the axes represent the contribution rate of the first (PC1) and second (PC2) principal components to the total amount of variation. **b** Heat map of the hierarchical cluster analysis. The *columns* indicate experimental groups of MSCs with addition of AAP (1 mM), and controls (0 mM). The *rows* show the normalized levels of each metabolite. The dendrogram for each heatmap shows the relatedness of the normalized metabolite level patterns. **c** Changes in carnitine expression in MSCs cultured in the presence of AAP. **d** Changes in various ribonucleotides in MSCs cultured in the presence of AAP. **e** Changes in metabolites associated with TCA in MSCs cultured in the presence of AAP. **p < 0.01

Metabolic changes caused by AsA addition were studied after adding 1 mM AAP to DMEM (10% FBS) for culturing P4 MSCs. After 1 day of culture, metabolic products were isolated and evaluated by mass spectrometry. Principal Component Analysis (PCA) clearly differentiated the AsA-supplemented group and the control group on the X-axis (Fig. 3a). In addition, hierarchical clustering analysis revealed that the abundances of metabolites in the AsA-supplemented group and the control group were reversed (Fig. 3b). AAP and AsA were both detected solely in the supplemented groups, and not in the control group (data not shown). We also observed an increase in carnitine, which participates in lipid metabolism in mitochondria and requires AsA when synthesized from lysine and methionine (Fig. 3c). We found marked increases in ATP, GTP, UTP, and CTP among metabolic products related to nucleotide metabolism (Fig. 3d). Furthermore, we noted a significant rise in glutathione, which has antioxidant properties (Fig. 3e). In addition, citric acid, fumaric acid, and malic acid, which are involved in the TCA cycle were increased in AAP-supplemented cells, suggesting mitochondrial activation (Fig. 3e).

### AsA reduces the expression of genes downstream of hypoxia inducible factor 1,alpha subunit (HIF1α)

**Fig. 4:**
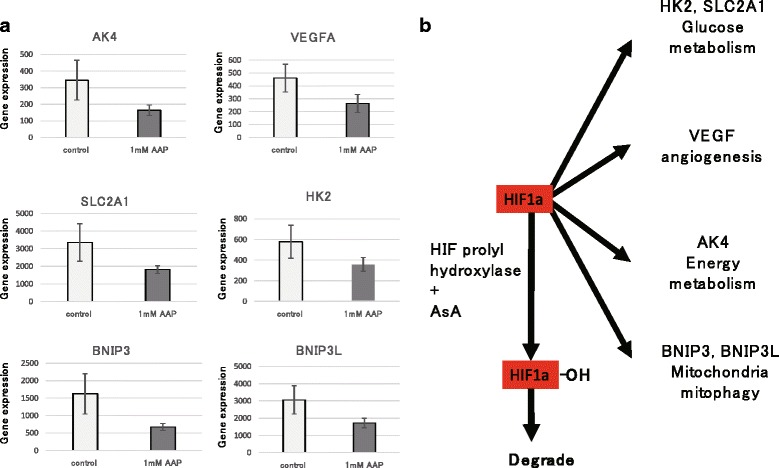
AAP changes the expression of genes downstream of HIF1α. **a** Genes downstream of HIF1α are downregulated in response to AAP addition according to SAGE. **b** Scheme representing the genes regulated by HIF1α according to function. **p < 0.01

SAGE revealed increased expression of genes relevant to cell proliferation and survival, while genes relevant to proliferation inhibition and cell death were downregulated in the AAP-supplemented groups (Table 1). Interestingly, multiple genes downstream of HIF1α had reduced expression. For example, we found a decrease in the expression of adenylate kinase 4 (AK4) (ratio = 0.45), which is involved in energy metabolism in the mitochondria, VEGFA (ratio = 0.57), which is involved in angiogenesis, hexokinase 2 (HK2) (ratio = 0.62), which is involved in the glycolytic system, and B-cell lymphoma 2 (BCL2)/E1B-19 kDa interacting protein 3 (BNIP3) (ratio = 0.41), which is involved in mitochondrial quality control (Fig. 4a, b).

**Table 1:**
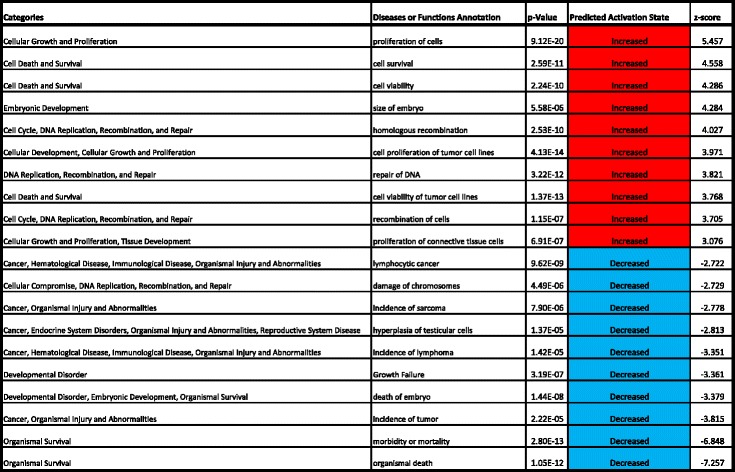
Disease or functional annotations exhibiting a significant change in MSCs by the addition of AsA

Metabolites showing significant upregulation are shown in *red* (*p* < 0.05), and those showing significant downregulation are shown in *blue* (*p* < 0.05)

### AsA promotes HIF1α breakdown induced by deferroxamine (DFO) in MSCs

**Fig. 5:**
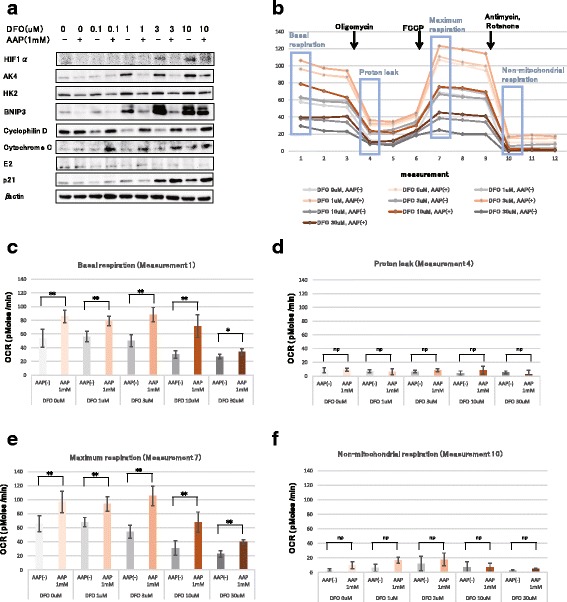
AAP antagonizes the hypoxia-mimetic state caused by DFO. **a** Western blot. Proteins were extracted after 2 days of culturing in media containing the indicated concentrations of DFO, with/without 1 mM AAP. **b** Effect of DFO and AAP on OCR. The OCR was measured using a flux analyzer before and after the administration of oligomycin (added before cycle 4), FCCP (added before cycle 7), antimycin A, and rotenone (added before cycle 10). **c** Basal respiration at cycle 1. **d** Proton leakage at cycle 4. **e** Maximal respiration at cycle 7. **f** Non-mitochondrial respiration at cycle 10

Because the SAGE results indicated that AsA decreases the expression of genes controlled by HIF1α, we used western blotting to evaluate protein expression after supplementing the medium with 1 mM AAP and DFO, which is an iron chelator that simulates hypoxia by suppressing HIF1α breakdown. HIF1α increased with DFO concentration and decreased with the addition of AAP. In addition, proteins induced by HIF1α, such as AK4, HK2, and BNIP3, increased in a DFO concentration-dependent manner, but decreased with AAP addition. Mitochondrial membrane proteins including cyclophilin D, cytochrome C, and E2 were downregulated by DFO addition, but were upregulated by AAP addition (Fig. 5a). Furthermore, when we studied oxygen consumption using a flux analyzer, we found that the oxygen consumption decreased in a DFO concentration-dependent manner, but increased when AAP was added (Fig. 5b). Moreover, AAP addition suppressed the decrease in OCR induced by DFO in terms of basic respiration (Fig. 5c) and maximum respiration (Fig. 5e). However, there were no significant changes in proton leakage (Fig. 5d) and non-mitochondrial respiration (Fig. 5f). Together, these results indicated that AAP addition activates mitochondria by promoting HIF1α breakdown.

### AsA affects DNA methylation in MSCs

It was recently reported that AsA is involved in epigenetic modification, especially DNA methylation. We used a beads array for DNA methylation analysis (Additional file 3: Figure S1). The percentage of differentially methylated probes was 0.29% (Additional file 3: Figure S1A). Among the differently methylated probes, 29.6% were hypermethylated and 70.4% were hypomethylated (Additional file 3: Figure S1B). A functional genome distribution of hyper- and hypomethylated probes is shown in Additional file 3: Figure S1C and D, respectively. Only hypermethylated sites were found in the 1500bp region upstream of the transcription start site (TSS). Significantly changed methylated probes were shown in Additional file 4: Table S1.

## Discussion

Numerous studies have reported various effects of AsA on cell division and proliferation. In cell culture in DMEM at 37 °C, AsA is rapidly oxidized; one study reported that the concentration was lowered by half after 1 day, and was negligible after 3 days [11]. Therefore, in this study, we used AAP, which exhibits low decomposition under normal culturing conditions, to evaluate the effects of AsA on proliferation and differentiation ability in MSCs, as well as metabolic changes via gene expression and metabolome analyses. Choi *et al*. have reported that MSC proliferation and differentiation were both promoted by the addition of 0.005–0.25 mM AAP, in a test range of 0–0.5 mM AAP [12]. Addition of high concentrations of AsA has been recently reported to promote oxidation and ATP starvation [13]. Therefore, we evaluated the effect of AAP up to the high concentration of 3 mM. When we added 0.1, 1.0, and 3.0 mM of AAP to MSCs at P4, all concentrations promoted proliferation to the same degree. In addition, when MNCs isolated from bone marrow were plated and cultured on a polystyrene culture plate to expand MSCs, AAP strongly promoted MSC proliferation to nearly the same extent at each concentration. When cells are isolated from bone marrow and plated/cultured (P0), the number of MSCs is extremely low, resulting in slow proliferation. Our results indicated that the addition of AsA is particularly useful for enabling faster MSC expansion.

Collagen is formed from intracellularly synthesized procollagen that is secreted from the cells, after removal of a portion of the peptide, converting it to tropocollagen. Collagen fibers are formed from tropocollagen by a hydroxylase, and hydrogen bonding of hydroxyproline in the collagen fibers results in the formation of a stable helical structure. AsA is known to be important as a cofactor of the hydroxylase that participates in the posttranslational modification of the collagen molecules [14], indicating that collagen participates in promoting proliferation. Thus, we compared proliferation in cells grown on a collagen-coated dish without AAP addition and in cells grown on a polystyrene dish with AAP addition. Addition of AAP in a polystyrene culture plate resulted in nearly the same cell count at 60 h after seeding as that observed in the collagen-coated dish without AAP addition (Fig. 2a), indicating that with regard to cell proliferation, adding AAP could be a substitute for collagen coating. In addition, proliferation increased in the collagen-coated dish until 24 h after seeding, while after this time point, the proliferation rate did not differ between coated and uncoated dishes. This suggests that collagen is important for MSC adhesion to the dish, but does not have a large effect on proliferation, and thus, that factors other than collagen affect proliferation.

There were no significant differences in the proliferation and collagen deposition of MSCs cultured in medium containing 0.1 mM, 1 mM, or 3 mM AAP. However, promotion of adipogenic differentiation by AAP also occurred in a dose-dependent manner. It has been reported that transcription factors such as PPAR-γ and C/EBPs are involved in this differentiation of adipogenic progenitor cells into adipocytes. As AsA, besides having an antioxidant effect, is a cofactor for various enzymes, it acts in a complex manner. The deep connection between AsA and differentiation has been reported based on the fact that it promotes collagen synthesis and promotes differentiation of iPS cells into cardiac muscle caused by the proliferation of cardiac progenitor cells mediated by the MEK-ERK1/2 pathway [8]. In addition, the induction NOX and eNOS by AsA, not its effect on collagen, is related to the promotion of differentiation [10]. Our SAGE data indicated an extremely low amount of NOX and eNOS expression in MSCs, and AsA did not clearly induce their mRNA expression. Thus, in this study, AsA seems not strongly involved in the induction of differentiation. However, interestingly, it has become clear in recent years that HIF1α plays an important role in controlling differentiation and maintaining stem cell morphology. The HIF1α subunit includes an oxygen-dependent degradation domain, in which two proline residues are hydroxylated by prolyl hydroxylases, which require AsA, Fe^2+^, and α-ketoglutaric acid as cofactors. The HIF1α protein is rapidly broken down in a proteasome-dependent manner as a result of the hydroxylated proline residues being ubiquitinated by E3 ubiquitin ligase. Thus, the intracellular quantity of HIF1α can drop sharply. For example, it has been reported that in the differentiation of hematopoietic cells, the strict regulation of differentiation by the quantity of HIF1α is important in maintaining hematopoietic stem cells through hypoxia [15]. Endosteal cells, which form a niche for hematopoietic stem cells, produce *Cripto*, which is known to be involved in ES cell differentiation and carcinogenesis in a HIF1α-dependent manner. Moreover, it has been reported that stimulation of glucose-regulated protein 78, which is localized at the surface of hematopoietic stem cells, successively activates *Akt* and HIF1α, maintaining hematopoietic stem cells [16].

As our results indicated that factors other than collagen have an effect on proliferation, we did a more detailed evaluation using metabolome analysis. ATP, GTP, UTP, and CTP all exhibited a significant upregulation. Furthermore, we found an increase in TCA cycle metabolic products and oxygen consumption due to AAP addition. Together, these results suggested the occurrence of mitochondrial activation. HIF1α is known to be intimately involved in the control of mitochondrial activation, which it suppresses by inducing pyruvate dehydrogenase kinase 1 and suppressing pyruvate dehydrogenase (PDH) activity, thus inhibiting acetyl CoA production from pyruvate. In addition, HIF1α is known to regulate mitochondrial elimination as it induces BCL2/BNIP 3, thus inhibiting the binding of Ras homolog enriched in brain (RHEB), which is important in the activation of the mTOR pathway, which induces mitophagy. In addition, the suppressed energy consumption resulting from HIF1α reduces gene translation efficiency by suppressing the mTOR pathway. Furthermore, intracellular AsA preferentially suppresses the HIF-1 transcriptional response by increasing HIF-hydroxylase activity [17, 18]. Our SAGE suggested that the HIF1α signal decreased, as indicated by the reduced expression in SLC2A1 (GLUT1) and HK2, which are involved in sugar metabolism, VEGFA, which is involved in angiogenesis, AK4, which is involved in mitochondrial energy metabolism, and BNIP3, which is involved in mitophagy, all of which are genes that are downstream of HIF1α. Furthermore, when 1 mM of AAP was added to cells to which DFO had been added, the expression of HIF1α and the downstream proteins was reduced. Thus, in MSCs too, AAP increases HIF1α hydroxylase activity, suppressing the HIF1a transcription reaction and leading to mitochondrial activation.

Besides HIF1α, another notable metabolic pathway related to mitochondrial activation promoted by AsA was carnitine synthesis. Carnitine is synthesized from lysine and methionine, and AsA is necessary as a hydroxylase coenzyme in this process. Carnitine is a component necessary for long-chain fatty acids to permeate the mitochondrial membrane. Fatty acid acyl-CoA is produced by acyl-CoA synthetase at the outer mitochondrial membrane, and acylcarnitine is produced by carnitine-palmitoyltransferase 1 present at the inner surface of the outer membrane. Acylcarnitine traverses the inner mitochondrial membrane through the action of carnitine-acylcarnitine translocase at the inner mitochondrial membrane, and is transported to the mitochondrial matrix. There, it is converted to acyl-CoA by CPTII at the inner mitochondrial membrane and is metabolized in the fatty acid beta-oxidation system. In this study, carnitine levels rose to 1.25 times when AAP was added, and thus, likely contribute to mitochondrial activation.

*2*-*Oxoglutarate*/*Fe*^2+^-dependent dioxygenases (2-OGDDs), which are enzymes that add a hydroxyl group to a variety of substrates and have been garnering attention in recent years, have AsA as a specific co-factor. Enzymes belonging to the 2-OGDD family are involved in collagen maturation, HIF hydroxylation, and carnitine synthesis, as well as in the demethylation of cytosine and histone [17, 18], which are processes we examined in this study. In AsA-deficient environments, such as in DMEM culture media, these pathways are suppressed. In particular, it is conceivable that HIF1α accumulation causes expression of downstream genes, causing a change to a hypoxia-mimetic metabolism, thereby exerting a strong effect on cell proliferation and metabolism. Previously, culturing MSCs in hypoxic conditions has been considered useful for proliferation, viability, colony formation, and suppression of aging [19–22]. Thus, culturing of MSCs without AsA unintentionally created hypoxia-mimetic conditions, which may be beneficial in terms of genomic stability and maintaining undifferentiated status. When there is no need to strongly promote proliferation or collagen production, it may not be necessary to add AsA. However, the presence of AsA results in culturing conditions closer to those in vivo, and adding AsA can be considered beneficial, particularly, when expanding from P0 or adipogenic differentiation of MSCs is necessary.

Much attention has been given to AsA-mediated DNA methylation and demethylation as epigenetic modifications [23]. Accordingly, we found changes in AsA-mediated DNA methylation, suggesting that AsA is important for proliferation and differentiation of MSC. It has been reported that deficiency of ascorbate disrupts the methylation-demethylation dynamics of DNA and histones, which can contribute to phenotypic alterations or even diseases [24]. Detailed AsA mediated epigenetic modifications in MSC remain to be elucidated.

**Fig. 6:**
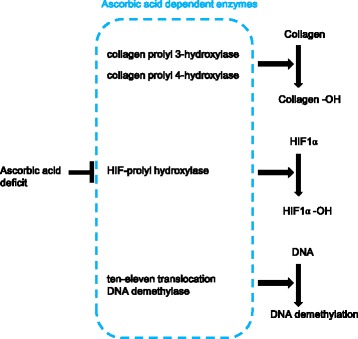
Proposed mechanism of the effect of ascorbic acid on mesenchymal stem cells. A deficit of AsA in the culture medium decreases the activity of ascorbic acid-dependent enzymes including collagen prolyl 3-hydroxylase, collagen prolyl 4-hydroxylase, HIF-prolyl hydroxylase, and ten-eleven translocation DNA demethylase. Collagen prolyl 3-hydroxylase and collagen prolyl 4-hydroxylases are crucial for the formation of the triple helix. HIF-prolyl hydroxylase catalyzes the posttranslational formation of 4-hydroxyproline in HIF1α proteins. Tet has been shown to convert the covalent epigenetic mark 5-methylcytosine to 5-hydroxymethylcytosine in DNA

Finally, we summarized the mechanism of the effect of ascorbic acid on mesenchymal stem cells in Fig. 6. A deficit of ascorbic acid in the culture medium decreases the activity of ascorbic acid-dependent enzymes. In future, further evaluation of the utility of AsA addition in various applications and of the effects of AsA on cells are desired.

## Conclusions

Our study indicated that the addition of AsA during MSC expansion leads to more efficient preparation of cells and is expected to enhance the application of MSCs in regenerative medicine. These are expected to be important findings for the future application of MSCs in regenerative medicine.

## Additional files

Additional file 1: Additional Methods. (DOCX 22 kb)

Additional file 2: **Figure S2.** Evaluation of differentiation potential of BMSCs with and without addition of AAP (1 mM). All differentiation procedures were induced using a Stem Cell Kit (R&D Systems) with mionor modifications and DMEM instead of αMEM. The reagents used for osteogenic and cholangiogenic differentiation included 0.05 mM AAP, respectively. (A) Adipogenic differentiation was evaluated by Oil Red O staining at 14 days after induction. (B) Adipogenic differentiation was evaluated by determining the PPARγ (peroxisome proliferator-activated receptor) expression level at 14 days after induction. (C) Osteogenic differentiation was evaluated by Alizarin Red staining at 14 days after induction. The reagent for osteogenic differentiation included 0.05 mM AAP. (D) Osteogenic differentiation was evaluated by determining the RUNX2 (runt-related gene 2) expression level at 14 days after induction. (E) Myogenic differentiation was evaluated by assessing multi-nucleation at 14 days after induction. The nucleolus was stained with Hoechst 33,342. (F) Myogenic differentiation was evaluated by determining the DMD (dystrophin) expression level at 14 days after induction. (G) Cholangiogenic differentiation was evaluated by Alcian Blue staining at 14 days after induction. The reagent for cholangiogenic differentiation included 0.05 mM AAP. (H) Cholangiogenic differentiation was evaluated by determining the ACAN (aggrecan) expression level at 14 days after induction. (EPS 29000kb)

Additional file 3: **Figure S1.** Changes in DNA methylation by AAP. A bead array for DNA methylation analysis was performed. (A) Summary of DNA methylation. (B) Differentially methylated regions. A functional genome distribution of hyper- (C) and hypo- (D) methylated probes. (EPS 2100 kb)

Additional file 4: **Table S1.** Differentially methylated genes (top 50). (XLSX 19 kb)

## Abbreviations

AAP: l-ascorbic acid 2-phosphate
AK4: Adenylate kinase 4
AsA: Ascorbic acid
BMMNCs: bone marrow-derived MNCs
BNIP3: Bcl-2/E1B-19 kDa interacting protein 3
DFO: Deferroxamine
HIF1α: Hypoxia inducible factor 1,alpha subunit
HK2: Hexokinase 2
MSCs: Mesenchymal stem cells
MNC: Mononuclear cells
OCR: oxygen consumption rate
