# Supplementary material for: Evaluation of the effects of ascorbic acid on metabolism of human mesenchymal stem cells
Source: Stem Cell Res Ther. 2018 Apr 6;9:93. doi: 10.1186/s13287-018-0825-1 (PMC5889584; doi:10.1186/s13287-018-0825-1)
Supplement: Supplementary file 1 — Additional Methods. (DOCX 22 kb) [file 13287_2018_825_MOESM1_ESM.docx]

**Additional Method**

**DNA methylation analysis**

DNA methylation was interrogated on an Illumina bead array. In brief, Standard Illumina protocol was used for labeling protocol (Cy5 and Cy3). Genomic DNA (500 ng) was treated with sodium bisulfite using the Zymo EZ DNA Methylation Kit (Zymo Research), according to the manufacturer's instructions. Bisulfite-converted DNA was whole-genome amplified (WGA), then enzymatically fragmented, and hybridized to Illumina Infinium MethylationEPIC BeadChip arrays according to the manufacturer's instructions. Polymer-coated chips were scanned on the iScan scanner (Illumina Inc.) with standard settings. Data preprocessing and methylation level extraction was performed using the Genome Studio software v2011.1 (Illumina Inc.) including the Methylation module.

**Analysis of BMSC differentiation potential**

BMSCs were cultured with each differentiation medium and evaluated for adipogenesis (Oil-Red O), osteogenesis (Alizarin Red), and chondrogenesis (Alcian Blue) by using a human MSC differentiation protocol (R&D) with minor modification [[1](#_ENREF_1)]. Myogenic differentiation was induced by same methods as previous reports [[2](#_ENREF_2)]. All differentiation procedures were induced using DMEM instead of αMEM to reduce the concentration of AsA. All samples were used 14 days after induction. We compared the expression of specific genes by using real-time PCR. Primers were purchased from Takara Bio (Otsu, Japan). The expressional level of PPARγ, RUNX2, and ACAN was normalized against GAPDH and shown as means ± standard deviation (SD). The expressional level of DMD was normalized against β-Actin.

1. Sasamoto T, Fujimoto K, Kanawa M et al. DEC2 is a negative regulator for the proliferation and differentiation of chondrocyte lineage-committed mesenchymal stem cells. **International journal of molecular medicine***.* 2016;38:876-884.

2. Park S, Choi Y, Jung N et al. Myogenic differentiation potential of human tonsil-derived mesenchymal stem cells and their potential for use to promote skeletal muscle regeneration. **International journal of molecular medicine***.* 2016;37:1209-1220.
